# A preliminary study on the application of PspA as a carrier for group A meningococcal polysaccharide

**DOI:** 10.1371/journal.pone.0218427

**Published:** 2019-07-10

**Authors:** Lichan Wang, Yajun Tan, Chen Wei, Huajie Zhang, Peng Luo, Shumin Zhang, Xiao Ma

**Affiliations:** DTaP and toxins division, National Institutes for Food and Drug Control, Key Laboratory of the Ministry of Health for Research on Quality and Standardization of Biotech Products, Beijing, China; Instituto Butantan, BRAZIL

## Abstract

This study aimed to explore the feasibility of pneumococcal surface protein A (PspA) as a carrier protein. Three recombinant pneumococcal surface proteins from three different clades were expressed by the prokaryotic expression system and conjugated to group A meningococcal polysaccharide (GAMP) to generate three polysaccharide-protein conjugates. The conjugates, unconjugated proteins, GAMP, and GAMP-TT vaccine bulk (used as positive control) were immunized into mice, and their immune effects were assessed by the methods of enzyme-linked immunosorbent assay (ELISA), flow cytometry (FCM), and serum bactericidal assay (SBA). The results showed that the polysaccharide-protein conjugates could produce higher levels of anti-GAMP IgG titers (*P* < 0.05), higher ratios of Th1/Th2 (*P* < 0.05), and higher levels of serum bactericidal activity (*P* < 0.05), compared with the unconjugated GAMP. The conjugation of PspAs to GAMP also enhanced the anti-PspA responses compared with unconjugated PspAs except for PspA3. In conclusion, the results indicated that the three PspAs were appropriate carrier proteins, as demonstrated by the characteristics of T-cell dependent responses to the GAMP, and might protect against group A of epidemic cerebrospinal meningitis.

## Introduction

Polysaccharide encapsulated bacteria, such as *Haemophilus influenzae* type b (Hib), *Streptococcus pneumoniae* (pneumococcus), *Neisseria meningitidis* (meningococcus), and group B streptococcus, cause a major proportion of diseases in early childhood. Capsular polysaccharide is a thymus independent antigen; immunization of infants and young children with this antigen does not induce high and long-lasting protective levels of serum antibodies. The success of the Hib conjugate vaccine highlighted the advantages of converting polysaccharides into T-dependent antigens by chemical conjugation to carrier proteins.

*Neisseria meningitidis*, a Gram-negative diplococcus known as the meningococcus, continues to be among the most important causes of bacterial meningitis worldwide. According to its surface-specific polysaccharide antigen, it can be divided into 12 groups, including A, B, C, X, Y, Z, E, W135, H, I, K, and L. Among them, polysaccharide conjugate vaccines based on groups A, C, Y, and W135 have been used in several countries [1–6]. However, the main prevalent strain in developing countries is group A [7]. According to reports from recent years, the annual incidence of meningococcal disease during epidemics reached 0.5–1 200 cases per 100 000 inhabitants. It is one of the serious public health problems worldwide [6–12].

The main carrier proteins used in commercial conjugate vaccines are the nontoxic mutant of diphtheria toxin (CRM197), diphtheria toxoid (DT), and tetanus toxoid (TT). With the increase in polysaccharide conjugate vaccines, the development of a new protein carrier is necessary to avoid immune tolerance, immune interference, or immune suppression due to repeated use of the same carrier [13, 14]. In addition, the disadvantage of the polysaccharide and conjugate vaccine is that their protection cannot cover all serotypes of the bacteria strains, such as pneumococcus. Serotype replacement limits their development and decreases their immune-protecting coverage. Thus, increasing the selection of a carrier protein or developing a new conjugate vaccine without serotype limitation is important and necessary in the future.

To solve the above-mentioned problems, pneumococcal surface protein A (PspA) was chosen for this study. PspA is found on the surface of all clinical isolates of *S*. *pneumoniae* [15] and interferes with opsonophagocytosis by blocking complement deposition on the bacterial surface [16, 17]. It has five domains: a signal peptide, α-helical and charged N-terminal domain, a proline-rich region, a choline-binding domain, and a short hydrophobic tail. PspA is relatively variable at the DNA and protein sequence levels. According to the sequences of α -helical region, PspAs are divided into six clades, which belong to three families [18, 19]. PspA family 1 comprises two clades (1 and 2), PspA family 2 comprises three clades (3, 4, and 5), and PspA family 3 comprises one clade (clade 6). Families 1 and 2 are expressed in more than 90% of strains [20]. Antibodies from different clades of the same family have relatively high levels of cross-reactivity and cross-protection, while the clades from different families have lower levels of these [21, 22]. Based on the structural diversity of PspA, it has been suggested that PspA-based vaccine should contain at least one clade from each of the two major families to elicit broad protection [23, 24]. In previous studies, PspAs have been used as carriers for pneumococcal and typhoid polysaccharides [25, 26]. Haiying Lin demonstrated the use of the carrier protein, pneumococcal surface protein A (PspA), conjugated with capsular polysaccharides, to provide effective and non-serotype-dependent protection. The CPS–rPspA conjugate not only induced CPS-specific protection but also provided PspA-specific cross-protection. In the study by Neha Kotharia, pneumococcal surface protein A was conjugated to Vi capsular polysaccharide from *Salmonella typhi* to make a vaccine against typhoid fever that had the potential to provide broad protection against *S*. *pneumoniae*.

In the present study, different clades of recombinant PspA protein (clade 1, clade 2, and clade 3) were prepared and conjugated to group A meningococcal capsular polysaccharide (GAMP) as carriers to make three conjugates, which not only prevented epidemic cerebrospinal meningitis but also had the potential to provide protection against *S*. *pneumoniae*. The immune effects, including humoral and cellular immune responses, were assessed after the immunization of mice. This study aimed to investigate the ability of the conjugates to prevent meningococcal infection. This prevention against pneumococcal infection will also be covered in the follow-up study.

## Materials and methods

### Ethics statement

All animal experiments were performed according to the Guide for the Care and Use of Laboratory Animals by the Ministry of Science and Technology of the People’s Republic of China and approved by the Institutional Animal Care and Use Committee of National Institute for Food and Drug Control (NIFDC). All mice were bred in specific pathogen free (SPF) individually ventilated cages (IVC) and given standardized diets at the animal facilities of NIFDC. After each experiment, the mice were euthanized by CO_2_ asphyxiation.

### Preparation of recombinant PspA proteins

*PspA* gene sequences of bacterial strain DBL6A (GenBank Association No. AF071805.1), RX1 (GenBank Association No. U89711.1), and EF3296 (GenBank Association No. AF071816.1) were obtained from GenBank and synthesized after codon optimization in accordance with *Escherichia coli* codon preference, so as to enhance their protein expression in the prokaryotic expression system. The synthetic truncated DNAs were 1143 bp for *PspA/ DBL6A*, 1122bp for *PspA/ RX1*, and 1431 bp for *PspA/ EF3296*, which belonged to clade 1, clade 2, and clade 3, respectively. Signal peptide and partial C-terminal sequences of these DNAs were excluded. The synthesized genes were cloned into plasmid pET-30a (+) vector with *Nde*I and *Xho*I restriction enzyme sites. To obtain an untagged protein, termination codons TAATAA were added before *Xho*I. Polymerase chain reaction (PCR) and enzyme digestion were used to identify the correction of the linkage. The expression plasmids were transformed into competent *E*. *coli* BL21 (DE3), cultured in Luria Broth (LB) medium at 37°C to an OD_550_ of 0.5. The protein expression was induced by adding isopropyl-D-1-thiogalactopyranoside (IPTG) to a final concentration of 1.0mM and incubating for 4 h. The following recombinant PspA molecules without His tag were produced: PspA/DBL6A (named PspA1) containing 375 amino acids, PspA/RX1 (named PspA2) containing 369 amino acids, and PspA/EF3296 (named PspA3) containing 471 amino acids. PspA1 and PspA2 proteins were sequentially purified by hydrophobic chromatography, ion exchange chromatography, and size exclusion chromatography; the PspA3 protein was purified sequentially by ion exchange chromatography, hydrophobic chromatography, and size exclusion chromatography. The three purified proteins were analyzed by sodium dodecyl sulfate polyacrylamide gel electrophoresis (SDS-PAGE). The antigenicity of the proteins was detected by Western Blot with a human serum of clinically diagnosed pneumonia. The human serum was taken from male hospital inpatients, aged 70 years, diagnosed with pneumococcal infection. The details were provided in previous studies [27].

### Preparation of conjugates

Cyanogen bromide (CNBr) activation method was used to conjugate three PspA proteins to GAMP respectively, which was completed in Beijing Lvzhu Biopharmaceutical Co. Ltd. (Beijing, China). The GAMP was concentrated to 5 mg/mL, and the final concentration of CNBr was 1 mg/mL to activate GAMP. The pH was adjusted with NaOH and maintained at 10.5. The mixture was stirred for 10 min at 20°C–26°C and Adipic Acid Dihydrazyde (ADH) was added to the final concentration of 0.25M. The pH was adjusted at 8.6 and maintained for 30 min. Then, the mixture was moved to a refrigerator (2°C–8°C) for overnight mixing to form GAMP–diacylhydrazide derivatives. The GAMP derivatives reacted with proteins in a 1:1 ratio with a reaction concentration of 2 mg/mL. The pH was adjusted and maintained at 5.6. Further, carbodiimide (EDAC) was added to a final concentration of 0.02M, and the pH was maintained at 5.6 for 90 min. The unpurified conjugates were obtained by ultrafiltration. The purification of the conjugates was carried out with Sepharose 4FF gel chromatography column, sterilized with 0.22-μm filter, and stored at 2°C–8°C. The ratio between proteins PspA and GAMP were determined based on the modified Lowry method and Phosphorus assay, respectively [28]. Table 1 lists the results of the conjugates. GAMP–TT (used as positive control) was provided by the Division of Respiratory Tract Bacterial Vaccine of NIFDC. GAMP was provided by Beijing Lvzhu Biopharmaceutical Co. Ltd.

**Table 1:** Ratios of polysaccharides to proteins for GAMP–PspA conjugates.

Groups	Polysaccharide content(μg/mL)	Protein content (μg/mL)	Ratio of polysaccharide/protein
GAMP–PspA1	57.3	91.4	0.63
GAMP–PspA2	63.1	100.8	0.63
GAMP–PspA3	121.5	195.6	0.62

### Animal immunization

Groups of 12 BALB/c female mice, weighing 16–18 g, were subcutaneously injected with each of the conjugate preparations or unconjugated controls. The dose of GAMP per injection was fixed at 2.5 μg with 0.5 mL. The protein dose per mouse was about 4.0 μg with 0.5 mL, which could be calculated according to the ratio shown in Table 1. Unconjugated proteins as control were injected at a dose of 5 μg per mice. All samples were free of adjuvants, and saline was used as a diluent. The mice received three doses on days 0, 14, and 28 and were bled by a retro-orbital puncture on days 13, 27, 35, and 180. The serum was separated and stored at –20°C for testing.

### Antibody detection

Anti-PspA and anti-GAMP IgG titers on days 13, 27, 35, and 180 were assayed by enzyme-linked immunosorbent assay (ELISA). Further, 3 μg/mL PspA and 3 μg/mL GAMP were coated overnight at 4°C in 96-well plates. The plates were washed with phosphate-buffered saline (PBS) containing 0.05% Tween 20 and blocked for 1 h at 37°C with PBS containing 10% BSA. Two-fold dilutions of mouse sera in PBS with the predetermined initial dilution were added to the plates and incubated at 37°C for 1 h. After the plates were washed, horseradish peroxidase (HRP)-conjugated goat anti-mouse IgG (Santa Cruz Biotechnology, Inc., Dallas, TX, USA) were added to the plates and incubated at 37°C for 1 h. OPD (Ameresco, Tully, NY, USA) substrate was used as the color agent. The reaction was stopped by adding 50 μL of 1M H_2_SO_4_ and read at 490 nm with a microplate reader. In addition, antibody subclasses against GAMP and PspA, IgG1 and IgG2a, were detected in the serum on the 35th day after immunization by the same ELISA method. HRP-conjugated goat anti-mouse IgG1 and IgG2a (Santa Cruz Biotechnology, Inc., Dallas, TX, USA) were used as the secondary antibody.

### Th1/Th2 detection by flow cytometry

Seven days after the third immunization (35th day), 1 mL of spleen cells (2×10^6^/mL) from mice in each group were co-cultured with 50 ng/mL phorbol myristate acetate, (PMA) (Sigma–Aldrich, MO, USA), 1 μg/mL ionomycin (Sigma–Aldrich), and 10 μg/mL brefeldin A (BFA) (BioLegend, CA, USA) for 4–6 h at 37°C in a humidified 5% air/CO_2_ incubator. The cells were washed with cell staining buffer and labeled with mouse CD3-PerCP and CD4-FITC antibody (BioLegend) for 20 min in the dark. Then, after the cells were fixed with fixation buffer and permeabilized with permeabilization wash buffer, they were labeled with mouse IFN-γPE and IL-4APC antibodies (BioLegend) for 30 min in the dark, washed, and suspended in cell staining buffer for flow cytometry (FCM) analysis.

### Bactericidal detection

Meningococcal strain and baby rabbit complement were provided by Lanzhou Institute of Biological Products Co. Ltd. (Gansu Province, China). Serum samples and rabbit complement for control were heat-inactivated for 30 min at 56°C for use in the assay. The serum and control samples were diluted threefold in 96-well plates for a total of 8 dilutions with initial fourfold dilution. Then, 10 μL of group A meningococcal strain was added to each well, and the plates were gently tapped to mix for 30 min. Further, 10 μL of complement or heat-inactivated complement was added to control the well or sample, respectively, which were cultured for 1 h at 37°C in a humidified 5% air/CO_2_ incubator. Next, 10 μL from eight serially diluted wells was taken onto the Todd-Hewitt yeast extract agar (THYA) plate and the plates were tilted immediately to make the liquid flow into one. The plates were maintained at room temperature for 20 min, transferred to a humidified 5% air/ 5% CO_2_ incubator, and kept at 37°C overnight. After overnight incubation, THYA plates were covered with triphenyltetrazolium chloride (TTC) agar and an automatic colony meter was used to calculate the number of colonies after agar solidification. Further, 50% bactericidal titer in the experimental group was calculated by comparing with that in the control group [29].

### Statistical analysis

SPSS 20.0 software (IBM, NY, USA) was used for statistical analysis. The significance of differences in various groups was assessed using a one-way analysis of variance (ANOVA). Pearson’s correlation coefficient was used to analyze the correlation between the results of serum bactericidal assay (SBA) and antibodies. Kolmogorov–Smirnov test was used to assess the normalization status of data. For all comparisons, a *P* value <0.05 was considered statistically significant.

## Results

### Production and purification of PspAs

**Fig 1:**
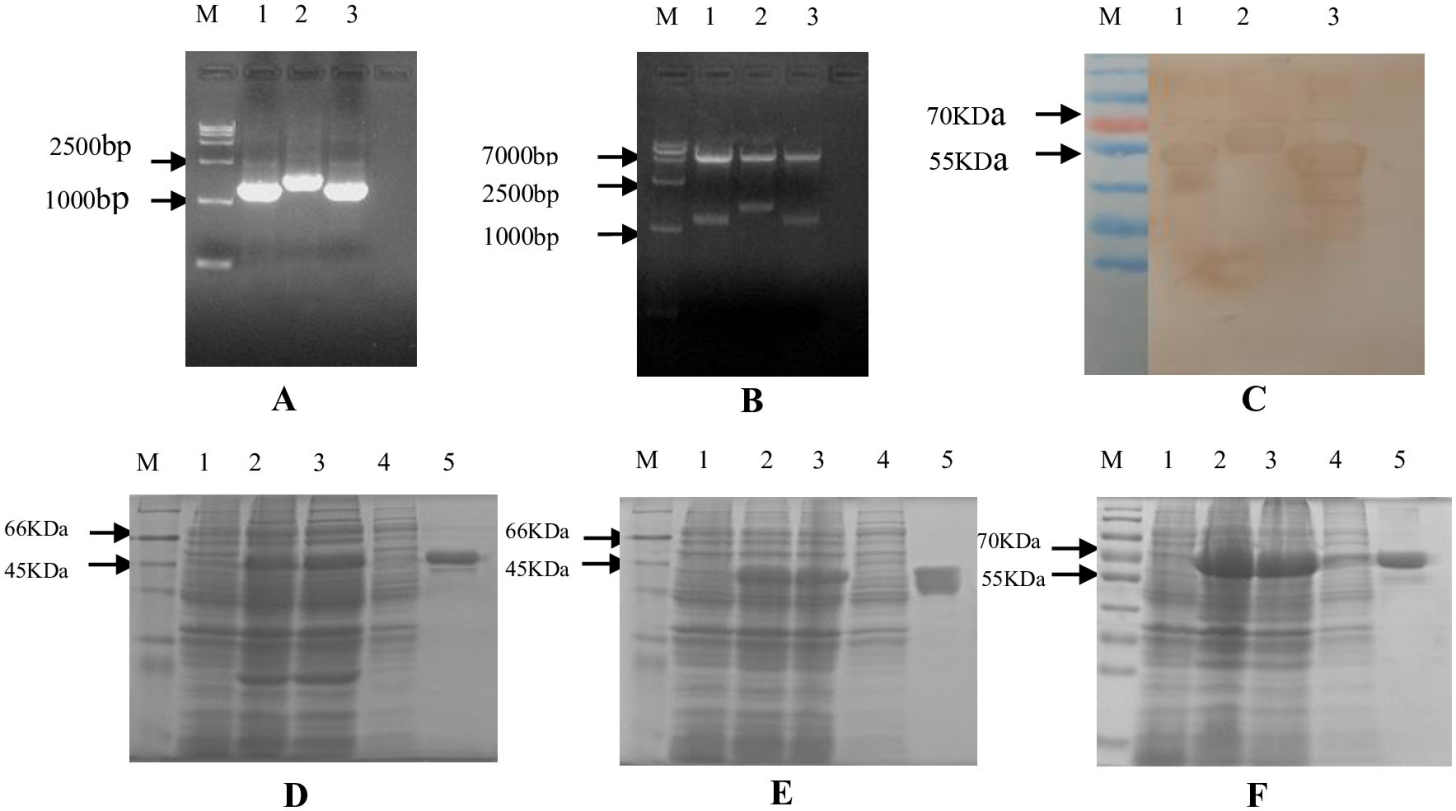
Preparation and identification of three PspA proteins. (A and B) Identification results obtained by PCR (A) and enzyme digestion (B) of three PspA expression plasmids. M: DNA Marker; Lane 1: PspA1 protein; Lane 2: PspA3 protein; Lane 3: PspA2 protein. (D–F) SDS-PAGE results for recombinant proteins PspA1, PspA2, and PspA3 in sequence. M: Marker; Lane 1: empty vector control; Lane 2: recombinant protein 4 h after induction with IPTG; Lane 3: supernatant of recombinant protein after sonication; Lane 4: precipitation of recombinant protein after sonication; Lane 5: supernatant of recombinant protein after purification. (C) Western blot analysis results of PspA proteins. M: Marker; Lane 1: recombinant protein PspA1; Lane 2: recombinant protein PspA3; Lane 3: recombinant protein PspA2.

The amplified products were 1000–2500 bp by PCR and enzyme digestion using expression plasmids as the template, which was consistent with the expected size (Fig 1A and 1B). Through three steps of chromatography, their purity reached 90% (Fig 1D and 1F). The results of Western blot analysis (Fig 1C) showed that the three proteins could react with the serum of patients with clinically diagnosed pneumonia, which indicated that the proteins had good antigenicity.

### Chemical and physical analysis of the conjugated GAMP

Three conjugates were synthesized: PspA1–GAMP, PspA2–GAMP, and PspA3–GAMP, which were prepared by cyanogen bromide activation method. The protein and polysaccharide contents were tested by the methods described in the Pharmacopoeia of the People’s Republic of China [24]. The ratios of polysaccharides to proteins were calculated according to their concentration (Table 1).

### Immunogenicity of the conjugated and unconjugated GAMP

**Fig 2:**
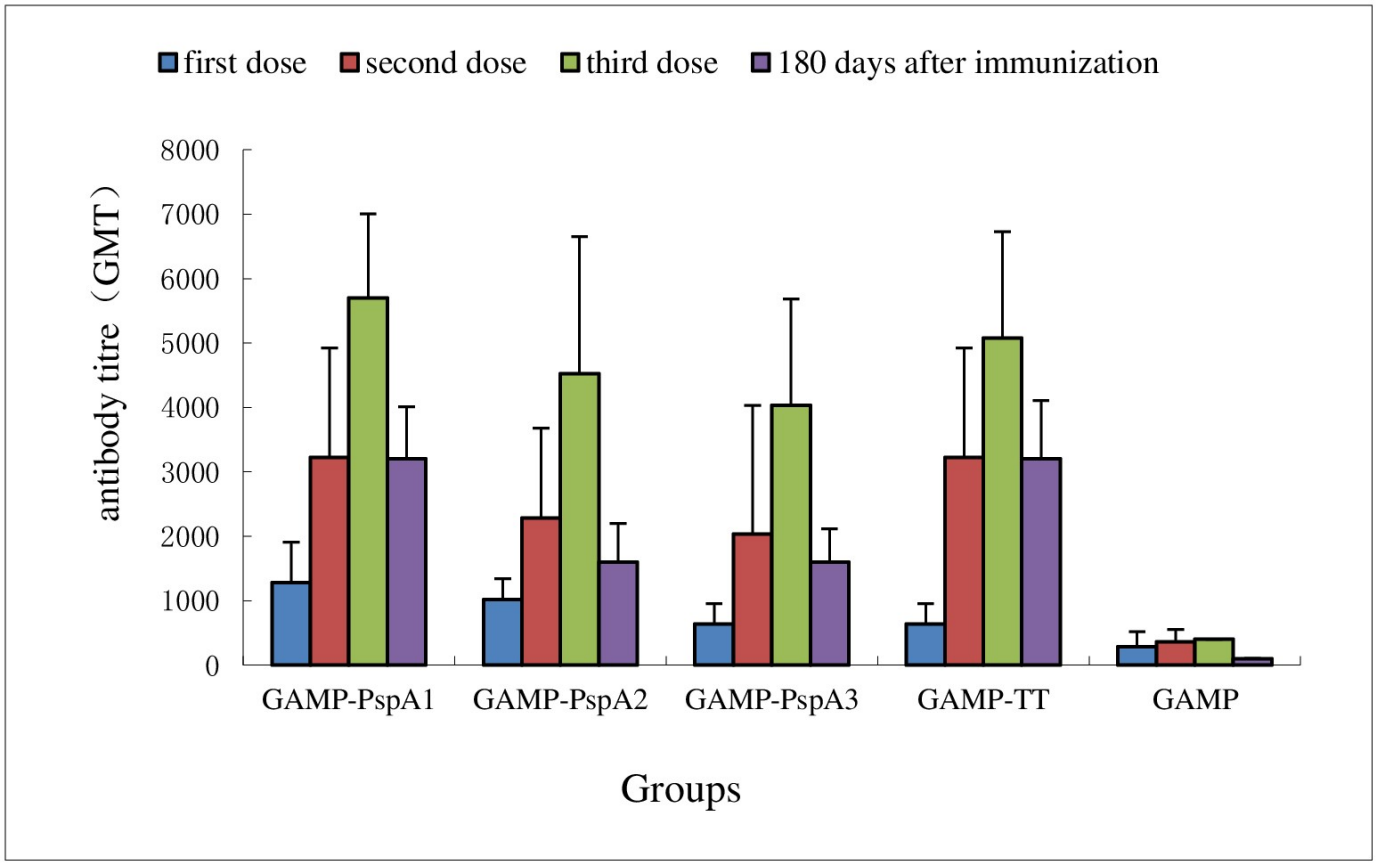
Results of anti-GAMP IgG antibody responses after the first, second, and third doses, and 180 days after the first immunization. The results were expressed as the reciprocal of geometric mean titer (GMT).

The anti-GAMP, anti-PspA, and anti-TT titers were calculated by comparing with that of PBS control mice sera and expressed as the reciprocal of geometric mean titer (GMT). The anti-GAMP responses are presented in Fig 2 (raw data see in S1 Table). All conjugates induced anti-GAMP responses after one dose, which increased with the second and third booster immunization and were significantly higher than that of unconjugated GAMP (*P* < 0.05). The anti-PspA (three clades) and anti-TT responses are presented in Table 3. The dose of PspA was almost the same for each conjugate, and the ratio of GAMP to PspA was very similar. The anti-PspA1 and anti-PspA2 responses following the first dose of conjugate were low; these responses were all boosted after the second and third doses and were significantly higher than that of unconjugated PspA1 and PspA2 (*P* < 0.05). The anti-PspA3 titer in the GAMP–PspA3 group was significantly lower after the third dose than that of unconjugated PspA3 (*P* < 0.05). For GAMP–TT conjugate, anti-TT titer was lower (dozens of times) compared with that of unconjugated TT either after the first immunization or after the booster immunizations, indicating that the conjugates inhibited the immunogenicity of TT. The IgG1 and IgG2a results of anti-GAMP and anti-protein are presented in Tables 2 and 3 (raw data see in S4 Table). The conjugate groups could enhance the production of anti-GAMP and anti-protein IgG2a subclasses compared with that of unconjugated GAMP and proteins. The serum in the GAMP group could hardly detect IgG2a subclasses under the same dilution conditions, indicating that the conjugates could induce class switch recombination to a specific antibody.

**Table 2:** Anti-GAMP IgG, IgG1, and IgG2a antibody levels of the conjugated and unconjugated GAMP after the 3rd dose (GMT) (95% confidence interval).

Groups	3rd dose (IgG)	3rd dose (IgG1)	3rd dose (IgG2a)	IgG1/IgG2a
GAMP–PspA1	5702(4494–7236)	5702 (44947236)	449 (69112)	13 (10–17)
GAMP–PspA2	4525(28337297)	4032 (23006764)	356 (64316)	11 (615)
GAMP–PspA3	4032(25315999)	3592(23615103)	400 (387404)	9(5–12)
GAMP–TT	5080(3598–7066)	4525(2959–6638)	400(387404)	11(615)
GAMP	400(387–404)	400(387–404)	/	/

Note: The results were expressed as the reciprocal of geometric mean titer (GMT). “/” indicates that no antibody was detected.

**Table 3:** Anti-protein IgG, IgG1, and IgG2a antibody levels of the conjugated and unconjugated GAMP after each dose (GMT) (95% confidence interval).

Groups	1st dose (IgG)	2nd dose (IgG)	3rd dose (IgG)	3rd dose (IgG1)	3rd dose (IgG2a)	IgG1/IgG2a
PspA1	45(29–63)	640(362–1023)	9051(-4804-33602)	3591(1943–6056)	251(47–585)	14(10–17)
GAMP-PspA1	202(125–299)	1280(575–2410)	14368(5974–41223)	3592(-2769-16767)	566(-974-2122)	6(2–12)
PspA2	113(73–165)	806(505–1199)	7352(-8654-32186)	4850(-10047-31712)	400(-494-2059)	12(5–16)
GAMP-PspA2	160(-8-460)	2560(1122–5489)	25600(16787–60543)	16127(9005–42459)	1600(1324–4023)	10(5–14)
PspA3	403(102–1229)	1140(-1487-6605)	51200(51080–51521)	40637(28792–56539)	3200(1438–6026)	13(8–16)
GAMP-PspA3	202(42–489)	2032(-643-7254)	7184(588–18610)	5702(4494–7236)	566(109–1488)	10(0–28)
TT	1016(419–2032)	10240(10227–10244)	40637(28792–56539)	40637(28792–56539)	3592(1942–6055)	11(0–15)
GAMP-TT	45(28–63)	113(73–165)	356(169–629)	356(169–629)	89(-18-283)	4(1–6)

Note: The results were expressed as the reciprocal of geometric mean titer (GMT).

### FCM of conjugated and unconjugated GAMP

**Fig 3:**
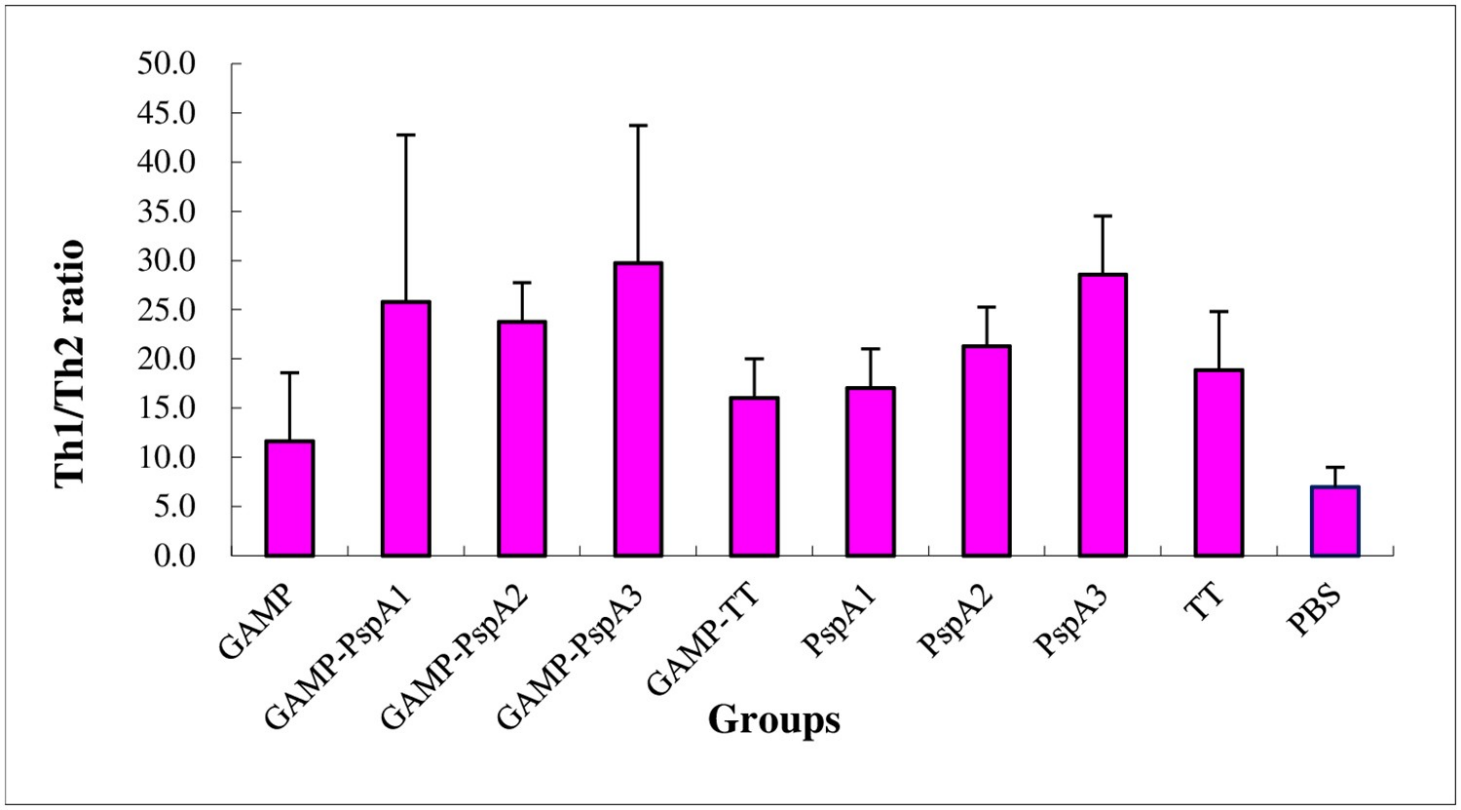
Results of FCM: Th1/Th2 ratio of the conjugated and unconjugated GAMP.

The test mainly detected the condition of immunized mice spleen cells after stimulating with nonspecific stimulants *in vitro*. IFN-γ and IL-4 cytokines were used as markers for Th1 and Th2 cell groups, respectively. The results of the Th1/Th2 ratio are shown in Fig 3 (raw data see in S2 Table). Compared with unconjugated GAMP and PBS control, the ratio of Th1/Th2 in the groups of polysaccharide–protein conjugates was significantly higher (*P* < 0.05). The ratio in the three GAMP–PspA conjugates was similar (*P* > 0.05), which was higher than that of in GAMP–TT conjugates (*P* < 0.05). No significant differences were found between the three unconjugated PspA groups and the three GAMP–PspA conjugates (*P* > 0.05).

### Bactericidal activity

**Fig 4:**
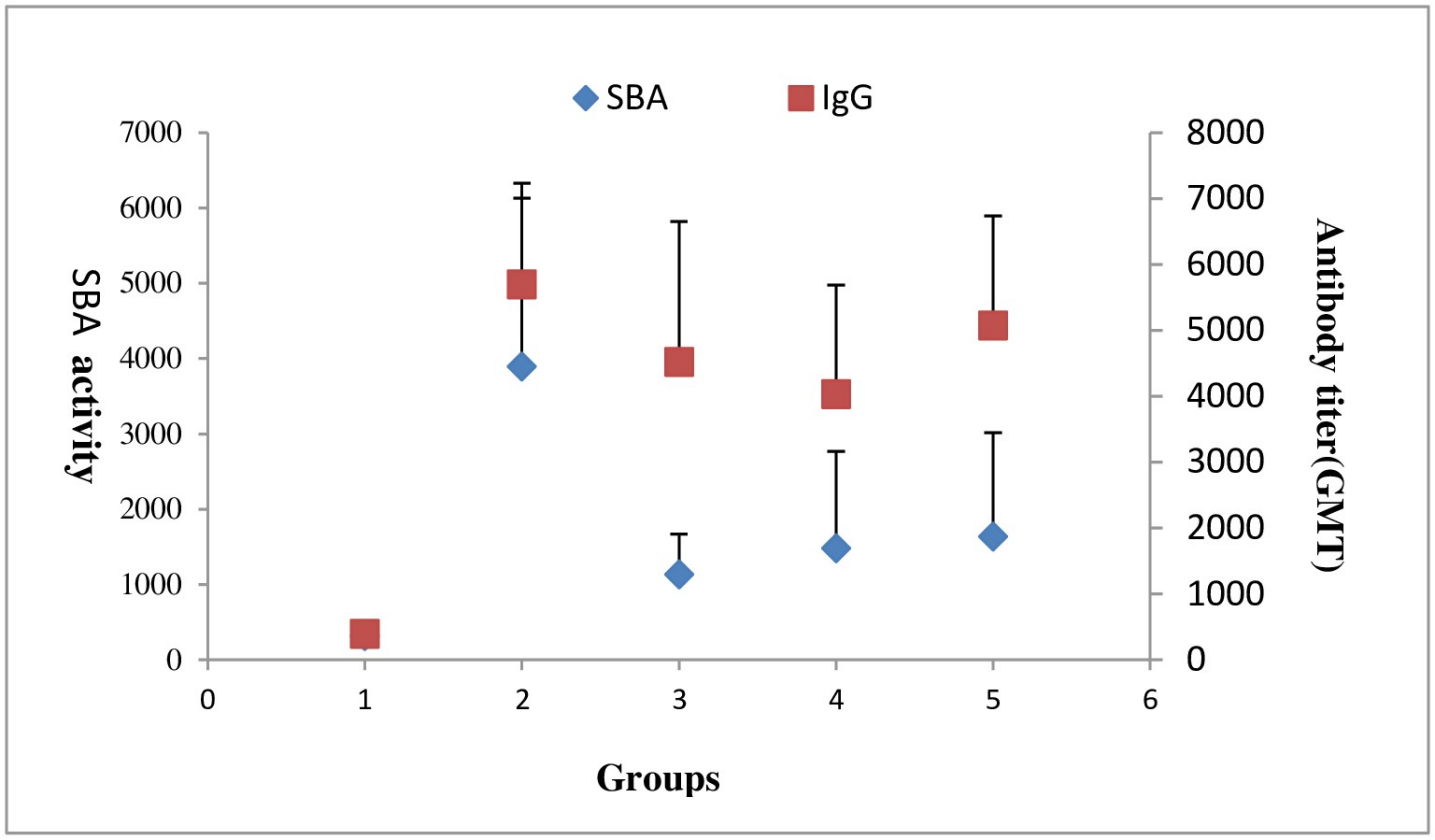
SBA activity and anti-GAMP antibody levels of conjugated and unconjugated GAMP, showing a consistent trend and positive correlation between the two groups (*R* = 0.604, *P* < 0.05). The results of SBA and anti-GAMP total IgG were expressed as the reciprocal of geometric mean titer (GMT). The 1–5 numbers on the horizontal axis represent Group GAMP, GAMP-PspA1, GAMP-PspA2, GAMP-PspA3 and GAMP-TT, respectively.

Serum bactericidal assay is a good test, which can directly reflect the potency of the meningococcal vaccine. SBA activities were expressed by geometric mean antibody titer. Fig 4 shows the results of the SBA activity and anti-GAMP antibody levels of conjugated and unconjugated GAMP (raw data see in S3 Table). Anti-GAMP IgG antibody responses in each group are also shown in Fig 4, the trend of which was nearly the same as that of SBA results. The Pearson’s correlation analysis was used to analyze the correlation between the results of SBA and antibodies, indicating that the SBA activity positively correlated with the antibody levels of anti-GAMP (*R* = 0.604, *P* < 0.05). The three polysaccharide–protein conjugates had higher SBA activity compared with that of unconjugated GAMP, and the difference was significantly different (*P* < 0.05).

## Discussion

Bacteria polysaccharide is a TI antigen that cannot stimulate T cells to produce cellular immune responses and cannot produce immunological memory. Therefore, a previous study conjugated carrier protein and polysaccharide by chemical methods, which changed the TI antigen properties of polysaccharides for TD antigen and resolved the above-mentioned challenges [30]. PspA was selected as a carrier protein of GAMP conjugate to provide broad cross-reactive protection against *S*. *pneumoniae*. The ratio of GAMP:PspA was about 0.63, which was similar to that for families 1 and 2.

The results showed that PspAs from both families 1 and 2 were appropriate as carrier proteins for preparing GAMP conjugates. The conjugation of PspA to GAMP resulted in a T-cell dependent response to GAMP, as demonstrated by boosting the antibody responses following the second and third doses, reflecting the induction of immunological memory. Anti-GAMP responses obtained after three doses of the GAMP–PspA conjugates were similar to those achieved with GAMP–TT conjugates. Unconjugated PspA from family 1 was poorly immunogenic compared with PspA conjugated to GAMP, demonstrating that the conjugation significantly increased the anti-PspA response. However, the conjugation of PspA from family 2 to GAMP did not increase the level of anti-PspA antibody, which was lower than that of unconjugated PspA3 (*P* < 0.05), especially after the third dose. The anti-TT response for GAMP–TT was similar to that for GAMP–PspA3; the immunogenicity of TT was inhibited by conjugation to GAMP. This might be due to the coverage of protein antigen epitope or due to protein changes in the process of combining polysaccharide to protein [31–34]. However, the exact reason needs to be further investigated.

For the analysis of Th1 and Th2, the ratio of the two (Th1/Th2) was expressed in the results of FCM. The high ratio indicated that the percentage of Th1 cells was high. The ratio of the three conjugates was higher than that of the corresponding unconjugated protein groups (*P* < 0.05), indicating that the conjugation groups induced higher levels of Th1 cells.

IgG1 and IgG2a are considered to be the markers of Th2 and Th1 responses, respectively. The results of antibody subclasses showed that the level of IgG1 was significantly higher than that of IgG2a, indicating that the experimental group had a strong ability to stimulate humoral immune response compared with cellular immune response. Despite this, IgG2a subclasses could still be detected, but the level of antibodies was low. In the present study, the ratio of IgG1/IgG2a was used for analysis. The lower the ratio, the higher the level of the induced IgG2a antibody, implying a stronger ability to induce cell immunity. The ratio of the three conjugates was lower than that of the corresponding unconjugated protein groups, indicating that the conjugated groups induced higher levels of Th1 cells. From this point of view, the results of IgG1/IgG2a were consistent with those of Th1/Th2. The above results indicated that the carrier protein conjugated with GAMP could activate the cellular immune response.

The SBA is one of the best methods for assessing the potency of meningococcal polysaccharide vaccine, which mainly detects the functional antibodies contained in the serum. It is the most direct method for evaluating the efficacy of meningococcal polysaccharide vaccine. It was proved to be associated with the protection of vaccine in the 1960s [35–38], and it has become the gold standard for evaluating the efficacy of the meningococcal vaccine. The results of SBA showed that the bactericidal activities of the conjugates were significantly stronger than that of unconjugated GAMP, and the potency of GAMP–PspA1 was higher than that of GAMP–TT (*P* < 0.05). Other PspA conjugates (PspA2 and PspA3) had no statistical differences compared with the GAMP–TT group (*P* > 0.05).

In conclusion, this study described a procedure for producing a GAMP conjugate using PspA as a carrier protein and evaluating the feasibility of PspA proteins. Humoral immune response, cellular immune response, and SBA assay were used to verify PspA proteins from both families 1 and 2, which could be appropriate carriers for preparing GAMP conjugates. The findings might promote the development of a new carrier protein and serve as a basis for the study of PspA protein as a carrier for other kinds of bacterial polysaccharide vaccine. However, the side effects of PspA and the potential of the conjugate immunization to prevent pneumococcal infection need further exploration.

## Supporting information

S1 Table Raw data for anti-GAMP IgG antibody responses. (XLSX)

S2 Table Raw data for FCM. (XLS)

S3 Table Raw data for SBA activity. (XLSX)

S4 Table Raw data for anti-protein IgG antibody responses. (XLSX)

## Abbreviations

ANOVA: one-way analysis of variance
ELISA: enzyme linked immune sorbent assay
FCM: flow cytometry
GAMP: group A meningococcal polysaccharide
GMT: geometric mean antibody titer
IFN-γ: fibronectin γ
IL-4: interleukin-4
NIFDC: national institutes for food and drug control
PspA: pneumococcal surface protein A
SBA: serum bactericidal assay
SFC: spot forming cells
TD: thymus dependent
Th: helper T cell
TI: thymus independent
